# Transforming watermelon *(Citrullus lanatus)* rind into durable superabsorbent hydrogels for enhanced soil water retention properties and adsorbs dye in water

**DOI:** 10.1016/j.heliyon.2024.e38656

**Published:** 2024-09-27

**Authors:** Bingqin Teng, Yuan Zhong, Jun Wu, Jiachen Zhu, Liqun Cai, Peng Qi, Zhuzhu Luo

**Affiliations:** a College of Resources and Environment, Gansu Agricultural University, Lanzhou, 730070, China; b State Key Laboratory of Aridland Crop Science, Gansu Agricultural University, Lanzhou, 730070, China; c Gansu Water-saving Agricultural Engineering and Technology Research Center, Lanzhou, 730070, China

**Keywords:** Hydrogel, *Citrullus lanatus*, Superabsorbent, Acrylic acid, Acrylamide

## Abstract

Innovative superabsorbent hydrogels were synthesized from watermelon rind (WR), an abundant agricultural waste. The process involved free radical polymerization of acrylic acid (AA) and acrylamide (AAm) with WR particles activated by ammonium persulfate (APS), resulting in (AA-co-AAm)/WR hydrogels with high equilibrium swelling capacities of 749 ± 32 g/g. Notably, after eight cycles, the WR hydrogel maintained 94.88 % of its initial swelling capacity, significantly outperforming the (AA-co-AAm) hydrogel without WR (13.80 % retention). This durability, combined with excellent water retention across various soil textures and high adsorption capacity for methylene blue (MB), underscores the WR hydrogel as a superior soil moisture conservation agent. This study marks a significant advance in recycling organic waste and enhancing water management in agricultural soils, demonstrating the potential for sustainable hydrogel development.

## Highlights

• This new type of watermelon rind hydrogel has higher water-absorbent properties (749 ± 32 g/g).
• The involvement of watermelon rind in the preparation of hydrogels can improve the service life of hydrogels.
• The novel hydrogel in this study significantly improves soil's water retention performance.
• Watermelon rind hydrogel adsorbed up to 1800 mg/g of methylene blue dye.

## Graphical abstract

**Figure:**
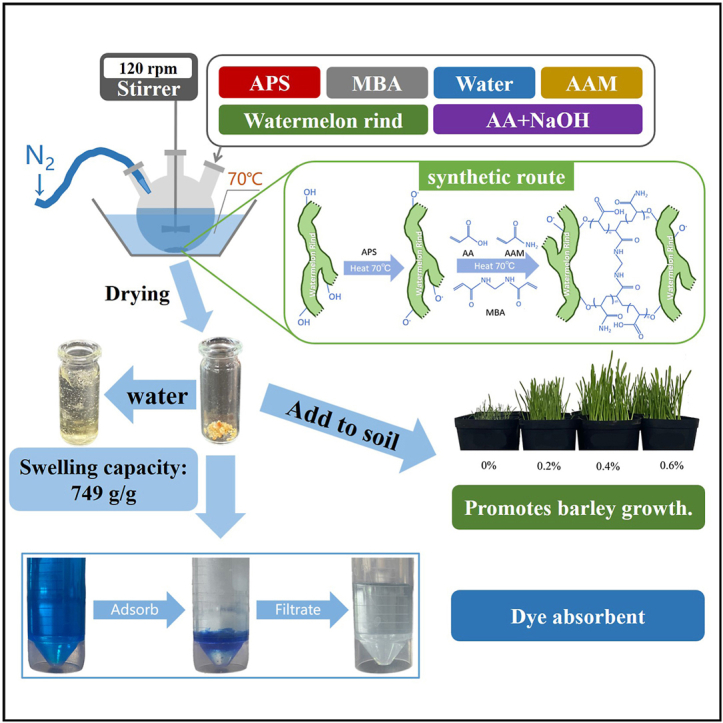

## 1 Introduction

Water scarcity, a critical global challenge, is intensified by climate change, population growth, and inefficient water use, especially in the agricultural sector. Agriculture, the primary consumer of freshwater resources, accounts for about 70 % of global withdrawals [1]. With the world's population on the rise, the demand for agricultural water is projected to surge significantly [2]. This demand is compounded by the uneven distribution of water resources, leading to acute scarcity in some regions while others enjoy abundance. Moreover, the low water holding capacity (WHC) of the soil, migration of water and nutrient into the deeper layer and excess surface runoff are equally responsible for water stress condition. The long-term drought can cause desertification, degradation of land, and affect the soil ecosystem [3]. Therefore, addressing water scarcity through sustainable water management practices is imperative to mitigate these harmful effects on crop production and ensure global food security [4,5]. The soil WHC is a crucial parameter for soil health, especially in adapting to extreme events like intensive rain and summer drought [6]. To enhance crop production in such environments, retaining a larger portion of rain or irrigation water in the topsoil is essential to prevent leaching or runoff. Superabsorbent hydrogel materials (with their unique three-dimensional) have shown promise in improving soil WHC by forming hydrophilic networks capable of absorbing and retaining water significantly beyond their original weight [7]. These hydrogels, characterized by their insolubility and ability to swell in water due to cross bonds, can act as water reservoirs in the soil, quickly absorbing and releasing water to plants as needed in the root zone. Incorporating superabsorbent hydrogel materials into soil management practices may effectively increase soil WHC, reduce water percolation losses, and enhance crop yield and water/fertilizer use efficiency [8].

In the late 1990s, Fredriksson and colleagues conducted molecular-level investigations on starch-based superabsorbent hydrogels, suggesting that the gelation process of pure starch primarily involves the entanglement and alignment of linear starch molecules [9]. These molecules, released from starch granules post-gelatinization, create a gel network by intertwining in a double helix manner during cooling and crystallizing in specific regions. The distinctive structure and performance attributes of starch-based superabsorbent hydrogel materials led to their rapid advancement and broad utilization across various sectors including medicine, hygiene, engineering, forestry, and horticulture [[9], [10], [11], [12], [13], [14]]. From 2005 onwards, researchers began incorporating water-absorbing functional groups or polymers (such as acrylic acid, acrylamide, isobutene maleic anhydride, and polyvinyl alcohol) into biodegradable natural polymers (like starch, carboxymethyl cellulose, chitosan, protein) to develop environmentally friendly biomass-based superabsorbent materials [[15], [16], [17]]. Subsequently, significant advancements were made in superabsorbent materials, with global production surpassing 2.3 million tons in 2015 [18]. These materials can be categorized into various types such as superabsorbent resins, fibers, films, fabrics, aerogels, and fabric-based aerogels, with applications spanning medical hygiene, daily essentials, wastewater treatment, safety measures, and agriculture [19,20]. Of particular note is the increasing emphasis on the use of superabsorbent hydrogels as soil water retention agents, as highlighted in recent studies [[21], [22], [23]].

Ratke et al. (2024) [24] evaluated the use of two hydrogels, one commercial and the other made from cashew (Anacardium occidentale) gum, to mitigate drought stress in soybean (Glycine max) cultivation. Results showed that the cashew gum hydrogel promoted a 12 % increase in seed protein content under drought stress. Additionally, the hydrogel application at 30 mg pot-1 improved soybean growth parameters and increased levels of essential nutrients like phosphorous (P), potassium (K), calcium (Ca), magnesium (Mg), and iron (Fe) concentrations. Similarly, AbdAllah et al. (2021) [3] examined the efficacy of three potassium-based super absorbent polymers (SAPs) in mitigating drought stress in maize under rainfed conditions in humid subtropical climates. The SAPs exhibited high water absorption capacity, reduced hydraulic conductivity, and enhanced soil WHC, resulting in reduced rainwater transport and increased soil water storage during simulated rainfall. Furthermore, in a pot study with corn, SAPs significantly reduced rainwater percolation, demonstrating their effectiveness in promoting plant growth and productivity. Although the current development of hydrogel is rapid, but there is still the problem that the service life of hydrogel is too short, and its water retention performance will decline rapidly when used in soil for a longer period of time.

Watermelon *(Citrullus lanatus)* is a widely cultivated crop globally, with China being a major producer, yielding approximately 60.4 million metric tons in 2022 alone [25]. The abundance of watermelon cultivation presents an opportunity for sustainable resource utilization, particularly in the context of developing eco-friendly materials. One such material of interest is watermelon rind (WR), the outer layer of the fruit that is often discarded as waste. However, there is a lack of research on utilizing WR to produce durable superabsorbent hydrogels, despite its potential inherent properties such as high cellulose content, biodegradability, and environmental friendliness. By repurposing WR into hydrogels, we not only reduce waste but also contribute to the development of sustainable and biodegradable materials with potential applications in agriculture, environmental remediation, and beyond. This approach aligns with the growing emphasis on utilizing renewable natural materials for various applications, including in the field of hydrogel synthesis. Moreover, the knowledge about the use of WR to develop superabsorbent hydrogels and its potential to improve barley germination under drought conditions needs to be highlighted. Therefore, in this study, a new class of super-absorbent hydrogels was synthesized using WR as an organic waste material source. This synthesis process involved graft copolymerization with acrylic acid (AA) and acrylamide (AAm), followed by chemical cross-linking using N, N′-methylenebisacrylamide (MBA). The investigation focused on the water-absorbing properties, water retention, and water-holding capacity of these hydrogels when combined with various soil types, as well as their reusability and impact on barley growth under drought stress conditions. It was hypothesized that WR can be effectively utilized to produce durable superabsorbent hydrogels with high water holding capacity and extended service life. Moreover, these hydrogels, synthesized through graft copolymerization and chemical cross-linking, are expected to enhance water retention and holding capacity when combined with different soil types. Furthermore, the hydrogels may positively impact barley growth under drought stress conditions, thereby contributing to sustainable resource utilization and improving soil water management in agriculture. Then the potential of hydrogel for adsorption of MB dye was explored by hydrogel adsorption test on methylene blue (MB) dye. The overall aim of this research was to develop a hydrogel material with high water holding capacity and a longer service life, which is crucial for reusing organic waste and enhancing the water holding and retention capacity of agricultural soils.

## 2 Materials and methods

### 2.1 Experimental materials

The chemicals utilized in this study, namely ammonium persulfate (APS), acrylamide (AAm), N,N' -methylenebisacrylamide (MBA), methylene blue (MB), acrylic acid (AA), and sodium hydroxide (NaOH), were procured from Shanghai Macklin Biochemical Science and Technology Joint Stock Company and were of analytical purity. The barley seeds used were of the Ganbeer 8 variety developed by the Gansu Provincial Academy of Agricultural Sciences. Given that the predominant soil textures in the Loess Plateau region include loam, sandy loam, sand, and saline soils, samples representing these textures were selected as experimental subjects. The essential characteristics of these diverse soil types are detailed in Table 1. Deionized water served as the testing medium throughout the experiments.

**Table 1:** Soil sources used in current experiment and their physiochemical properties.

No.	Soil source	Sand (%)	Silt (%)	Clay (%)	Soil texture	pH	SOC (g/kg)	Soil Capacity g/cm^3^
1	Procurement in Lanzhou City, Gansu Province	46	41	13	Loam	7.86	271.21	0.552
2	Collected at Lijiaobao Village, Dingxi City, Gansu Province, China	63	27	10	Sandy Loam	8.36	18.42	1.732
3	Collected at Jinsha Village, Anning District, Lanzhou City, Gansu Province	92	1	7	Sand	8.16	0.74	1.628
4	Collected at Jiulongjiang Forest Farm, Ganzhou District, Zhangye City, Gansu Province	88	11	1	Sand (saline)	8.94	7.03	1.586

### 2.2 Synthesis of hydrogels

**Fig. 1:**
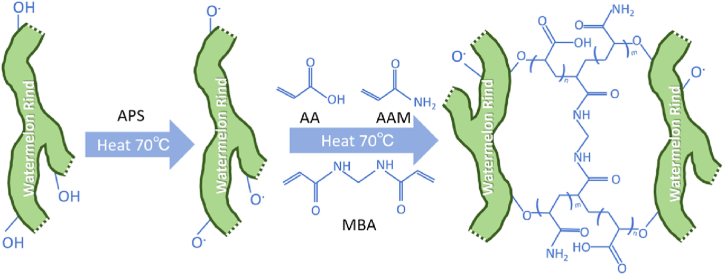
Roadmap for the synthesis of (AA-co-AAm)/WR hydrogels. Note: AAm is acrylamide; AA is acrylic acid; MBA is N, N′-methylene bisacrylamide; APS is ammonium persulfate.

The watermelon rind used in this study was collected from Huining, Gansu, China. The watermelon rind serves as a valuable source of cellulose and pectin, providing natural polymer materials suitable for hydrogel preparation. The initiation of free radical polymerization reactions was facilitated by ammonium persulfate (APS), a commonly used initiator. The oxidation of cellulose and pectin within the watermelon rind produced compounds containing carboxyl and hydroxyl groups. Upon heating the monomers acrylic acid (AA) and acrylamide (AAm) to specific temperatures (70 °C), free radical polymerization occurs, resulting in the formation of polyacrylic acid and polyacrylamide. N, N′-methylenebisacrylamide (MBA) acts as a crosslinking agent, creating a three-dimensional network structure within the polymer chain, essential for hydrogel formation. The synthetic pathway for the (AA-co-AAm)/WR hydrogel is illustrated in Fig. 1. The total mass of hydrogel prepared in this study was 576g [15,26].

The synthesis procedure for the (AA-co-AAm)/WR hydrogel involved the following steps: A specified amount of watermelon rind (WR) was mixed with deionized water to create a homogenous slurry by sample speed homogeniser (SpeedMill Plus, Jena, Germany), forming a 7.5 wt % aqueous suspension of watermelon rind, which was then transferred to a three-necked flask. Nitrogen gas was introduced into the flask for 30 min, and the water bath was heated to 70 °C. Ammonium persulfate (APS) was added in a predetermined quantity, followed by stirring for 30 min. Subsequently, specified amounts of acrylamide (AAm), N,N′-methylenebisacrylamide (MBA), and acrylic acid (AA neutralized to 40 % by NaOH aqueous solution) were added, along with the remaining deionized water, as indicated in Table 2 to achieve the desired wt % of WR and each reagent. The mixture underwent continuous heating until gel formation occurred. After gel formation, the samples underwent three washes with ethanol to remove any unreacted monomers, followed by three additional washes with ultrapure water to eliminate residual ethanol. Finally, the samples were dried at 40 °C in an oven and subsequently ground into a powder for further analysis [15].

**Table 2:** Reagent dosage table.

No.	WR (wt %)	AAm (wt %)	AA (wt %)	MBA (wt %)	APS (wt %)
W-1	5	3	7	0.05	0.2
W-2	5	3	7	0.025	0.2
W-3	5	3	7	0.1	0.2
W-4	5	5	5	0.05	0.2
W-5	5	7	3	0.05	0.2
CK-2	0	3	7	0.025	0.2

Note: WR is watermelon rind; AAm is acrylamide; AA is acrylic acid; MBA is N, N′-methylene bisacrylamide; APS is ammonium persulfate; The addition of acrylic acid needs to be neutralized with a 40 % aqueous sodium hydroxide solution; The W-1 to W-3 treatments were to vary the weight percent of MBA (0.025–0.1 wt %).The W-1, W-4, and W-5 treatments were to vary the ratio of AAm to AA (from 3:7–7:3); CK-2 was based on W-2 without the addition of watermelon rind.

**Fig. 2:**
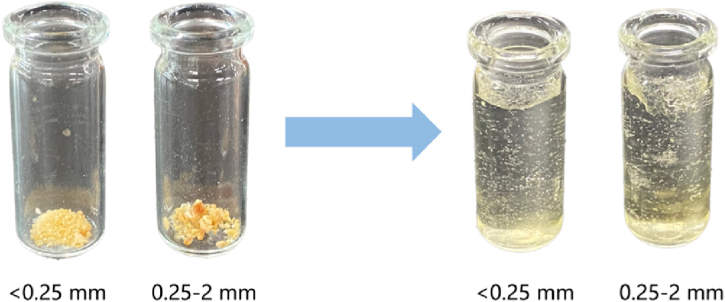
Before and after water absorption of (AA-co-AAm)/WR hydrogels of different particle sizes.

The powdered hydrogel samples were sieved through 0.25 mm and 2 mm sieves, and <0.25 mm and 0.25–2 mm particle sizes were screened as materials for subsequent experiments. The material numbers for <0.25 mm are shown in Table 2 The materials with 0.25–2 mm particle size were numbered with "-max" to differentiate them from each other (For example, the 0.25–2 mm grain size of the W-2 treatment will be expressed as W-2-max). The morphology of the hydrogel before and after water absorption is shown in Fig. 2.

With regard to the potential toxicity of polyacrylic acid and polyacrylamide hydrogels to crops, based on available research and information, both materials are generally considered biocompatible, especially polyacrylic acid because it contains a high number of hydrophilic carboxyl groups, and its monomer acrylic acid is harmless and responsive to changes in pH in the environment. However, the acrylamide monomer is considered toxic and carcinogenic in some cases, but in hydrogels, where acrylamide is usually present in a polymerized form, its toxicity can be greatly reduced [27].

### 2.3 Characterization of hydrogel

The Fourier transform infrared spectra (FT-IR) of the samples were measured in the wavelength range of 400–4000 cm^−1^ using an FT-IR spectral analyzer (Thermo Fisher Nicolet iS50, Thermo Fisher Scientific) by rapidly pressing the powders at a pressure of 10–15 MPa. The surface morphology of the samples was observed by gold spraying under a scanning electron microscope (SEM) (JEOL S-3400N, HITACHI, Japan) with a thickness of 53 mm and a voltage of 10 kV. Thermogravimetric analysis (TGA) of the samples was carried out in the temperature range of 25–900 °C at a ramp rate of 10 °C per minute in a nitrogen atmosphere using a mettler TG-DSC 3+ thermogravimetric analyzer from METTLER TOLEDO. Morphology, topography, and surface roughness were explored using a Bruker atomic force microscope (AFM) from Germany. Under room temperature conditions, a triaxial closed-loop scanner facilitated non-contact dynamic mode imaging [28,29].

### 2.4 Water absorption and retention test of hydrogel

(1) 0.05 g of hydrogel material from different treatments were loaded into pre-weighed tea bags respectively (repeated three times). (2) The tea bags were soaked in deionized water at room temperature for a period of time. (3) At 5, 10, 30, 60, 120, 240, 480, 720, and 1440 min, respectively, the tea bags were removed and the water hanging outside the tea bags was drained with filter paper and then weighed. (4) Obtain the water absorption curves of hydrogels at different times.

The swelling ratio Q and equilibrium swelling ratio Qeq were calculated by the following equations:

(Eq.1) $$Q=\frac{\left(W-W_{0}\right)}{W_{0}}$$

(Eq.2) $$Qeq=\frac{\left(Weq-W_{0}\right)}{W_{0}}$$

where $W_{0}$ is the weight of the dried sample, *W* is the weight of the swollen sample, and *Weq* is the weight of the swollen sample after reaching equilibrium.

The tea bags were then removed and placed at room temperature (temperature: 20 °C; humidity: 20 %). The weight of the hydrogel was recorded daily and continued for 14 days. The water retention curve of the hydrogel was finally obtained. The water retention performance of hydrogels was calculated by weighted summation of *Q* of each day, which was used for correlation analysis, structural equation model, and significance calculation using random forest model. The specific formula is shown as follows:

(Eq.3) $$Retention=\sum_{t=1}^{14}t\times {Qeq}_{t}$$

Where *t* is the time (days), ${Qeq}_{t}$ is the equilibrium swelling ratio on day *t*.

### 2.5 Hydrogel reusability studies

The hydrogel in 2.4, which has been swollen by absorption of water was dried in an oven at 60 °C to a constant weight and then immersed in water until the weight no longer changes. This was repeated, and the equilibrium swelling ratio (Qeq) of the hydrogels was recorded eight times to obtain the hydrogel reusability curve. The reusability performance of the hydrogel is defined as the ratio of Qeq at the eighth use to Qeq at the first use, utilized for correlation analysis, structural equation modeling, and significance calculations using a random forest model. The specific formula is as follows:

(Eq.4) $$Reusability=\frac{{Qeq}_{8}}{{Qeq}_{1}}$$

Where: Qeq_8_ is the Qeq value of the 8th use of the hydrogel and Qeq_1_ is the Qeq value of the first use of the hydrogel.

### 2.6 Study on the water absorption properties of hydrogels with different solution pH

The equilibrium swelling ratio (Qeq) of the hydrogel materials (W-2) was determined by placing them in solutions of pH 2, 3, 4, 5, 6, 7, 8, 9, 10, 11, and 12 (The pH in the solution is adjusted using NaOH and HCl). The Qeq curves of hydrogels at different pH were finally plotted.

### 2.7 Study on the effect of hydrogel on water holding and water retention properties of different soils

After adding 0 %, 0.2 %, 0.4 % and 0.6 % of the WR hydrogel (W-2) to different soils and fully wetting the soils, the water holding and water retention rates were determined and the curves were plotted based on the data to characterise the effect of different soils on the water holding and water retention rates of different soils after the addition of hydrogel. The specific method was as follows:

Fifteen grams of experimental soil were taken and mixed with 0g, 0.03g, 0.06g, and 0.09g of samples respectively. These mixtures were added to the flower pots, and three replicates were set for each group. Then, the experimental materials (flower pots + mixed samples) were placed into deionized water for one day. After 24 h, the materials were taken out, and weighed to obtain *W1*. Subsequently, they were placed at room temperature, and their weight Wt was measured every day to observe their changes until Wt remained essentially unchanged. A water retention curve was plotted based on the collected data.

(Eq5) $$Wh\%=\frac{\left(W1-W0\right)}{Ws}\times 100\%$$

(Eq.6) $$Wr\%=\frac{\left(Wt-Wdry\right)}{\left(W1-Wdry\right)}\times 100\%$$

Where: *Wh*% is the water-holding property; *Wr*% is the water-retaining property; *Ws* is the weight of soil + sample; *W0* is the weight of pots; *W1* is the weight of sample + pots + soil + water, after one day's immersion; *Wt* is the weight obtained by weighing at room temperature on day t (sample + pot + soil + water); *Wdry* is the weight when *Wt* stabilises.

### 2.8 Study on the water absorption of hydrogels in different salt ion solutions

To investigate the effect of salt ions on the water absorption rate of hydrogel and to derive the water absorption of hydrogel to different salt ion solutions at different periods. This was done by placing the hydrogel (W-2) into 0.9 % NaCl, CaCl_2_, and KCl solutions, respectively, and recording the swelling ratio of the hydrogel materials at 1-h intervals over 8 h.

### 2.9 Studies on the effect of hydrogel on barley germination under drought stress

In order to investigate the effect of added hydrogel (W-2) on barley germination under drought stress, we used the following methods:

Weighed 30g of potting soil, added 0 %, 0.2 %, 0.4 %, and 0.6 % of hydrogel, and set three replicates for each group. Barley seeds were rinsed three times with water and soaked for 10 h. After seed imbibition (just a little white color at the end of the seed, also called seed pre-germination), the seeds were planted into the potting soil with hydrogel added (3 g of seeds per pot) and placed into the incubator with the temperature set at 20 °C and the humidity as indoor humidity (about 20 %). The water treatment was set to 35 % Field Water Capacity (FWC). After seed germination, light was added to the incubator, barley growth was observed, photographed, and recorded. After 7 days, 12 beads of seedlings were randomly selected from each treatment to measure their plant height and aboveground part weight.

### 2.10 Adsorption test for methylene blue (MB) dye

Preliminary literature review and pilot experiments guided the selection of optimal conditions for the study. It was determined that 2, 4, 6, 8, 10, 12, 14, 16, 18, and 20 mg of hydrogel, respectively, immersed in 25 mL of 480 mg/L methylene blue (MB) solution (pH = 8), would best illustrate the impact of varying hydrogel concentrations on MB dye removal efficiency [30]. Then it was rotated on a thermostatic shaker at 200 rpm for 12 h at 25 °C. Then the MB concentration was determined colorimetrically in a UV spectrophotometer (UV-1700S, Macylab Instrument, China) at 664 nm. The dye removal (*R*, %) was calculated using the following equation [31].

(Eq.7) $$R=\frac{C_{0}-C_{e}}{C_{0}}\times 100\%$$

Where *C*_*0*_ and *C*_*e*_ are the MB concentrations (mg/L) before and after the adsorption test, respectively.

5 mg of hydrogel was placed into MB solutions (pH = 8) with concentrations of 5, 10, 20, 30, 40, 60, and 100 mg/L, respectively [30]. The MB concentration was measured after the same treatment as above. The adsorption capacity (*q*_*e*_) of the hydrogels was calculated using the following equation and fitted using the Langmuir isothermal adsorption model [31].

(Eq.8) $$q_{e}=\frac{C_{0}-C_{e}}{m}\times V$$

(Eq.9) $$\text{Langmuir}\;\;\text{model}：y=\frac{q\cdot x}{k+x}$$

Where *V* and *m* are the volume of MB solution (L) and the weight of hydrogel (g), respectively. *q* and *k* are the maximum adsorption amount and the degree of curve bending, respectively.

### 2.11 Data analysis

Data were organized using Excel 2019, one-way ANOVA using SPSS 23.0 statistical software, plotted using Origin 9.0, significance calculations using Random Forest Model using Python 3.12, and structural equation modeling using Amos26.

## 3 Results

### 3.1 FT-IR analysis

**Fig. 3:**
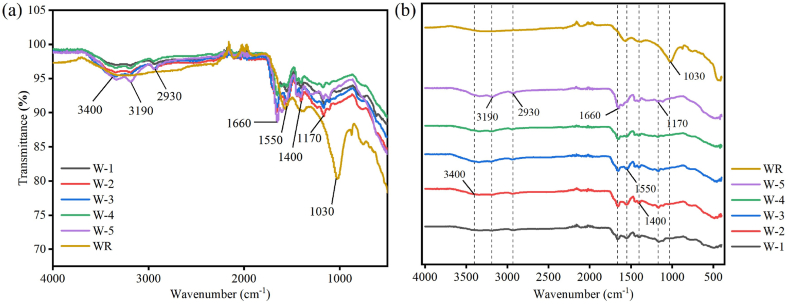
FT-IR spectra of different treated hydrogels; line graph (a), stacked graph (b).

Fig. 3 shows the FT-IR spectra of WR, W-1, W-2, W-3, W-4, and W-5 superabsorbent hydrogels. All hydrogel samples show similar spectral profiles. Among them, the characteristic absorption peaks of hydrophilic and hydrophobic functional groups indicate their significant contribution to the water absorption properties. The relatively weak (broad) bands produced by the bending vibrations of the amide group at 3400 cm^−1^ and 3190 cm^−1^ indicate the presence of hydrophilic amide groups [17]. The absorption peaks at 2930 and 1660 cm^−1^ are attributed to the stretching vibration peaks of N-H [27,[32], [33], [34]]. The band in the spectrum located at 1660 cm^−1^ is generated by the carboxylate group stretching vibration of the amide, the so-called amide I band. The peaks at wavelengths 1550 and 1400 cm^−1^ belong to COO- (symmetric) and COO- (asymmetric) stretching vibrations, respectively. The absorption peak at 1170 cm^−1^ attributed to the asymmetric oxygen bridge (C-O-C). The absorption peak at 1030 cm^−1^ attributed to the C-O stretching vibration in the alcohol hydroxyl group of watermelon rind was almost invisible after the reaction.

### 3.2 SEM analysis

**Fig. 4:**
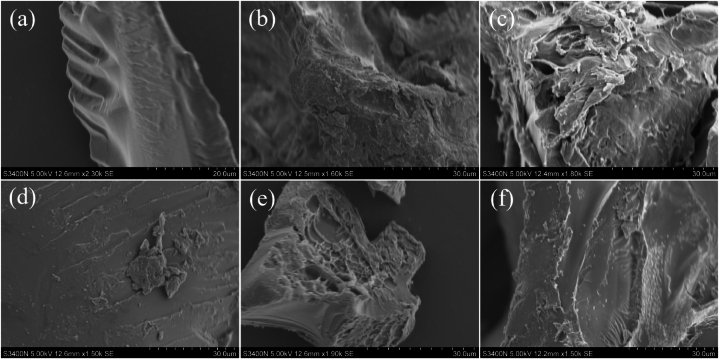
SEM images of CK-2 (a), W-1 (b), W-2 (c), W-3 (d), W-4 (e), W-5 (f).

Watermelon rind can be polymerized by free radical polymerization to form hydrogels with a three-dimensional network structure. Scanning electron microscopy (SEM) analysis of the superabsorbent hydrogels allowed a detailed view of the morphology of the hydrogel particles, leading to a comprehensive understanding of their structural features and water absorption capacity. Fig. 4 shows the surface morphology of CK-2 (a), W-1 (b), W-2 (c), W-3 (d), W-4 (e), W-5 (f). Among them, the surface characteristics of CK-2 were completely different from those of the hydrogel with watermelon rind added. The surface of the hydrogel with added watermelon rind is rough and layered, while the surface of CK-2 is smooth and compact with no air gaps. This is because watermelon rind is rich in cellulose pectin, etc [35]. By comparing the hydrogels with the addition of watermelon rind, it was found that the texture level on the surface of W-3 was weaker than the other four groups. This may have been caused by the addition of more cross-linking agents (MBA) to W-3.

### 3.3 AFM investigation

**Fig. 5:**
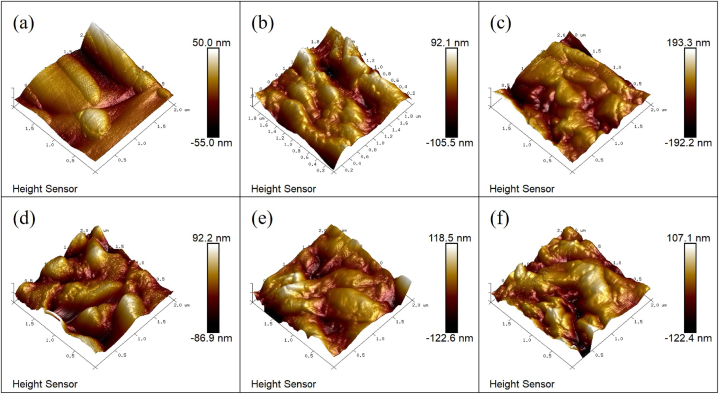
AFM images of CK-2 (a), W-1 (b), W-2 (c), W-3 (d), W-4 (e), W-5 (f).

An atomic force microscopy (AFM) investigation was conducted, elucidating three critical parameters: topography, height, and roughness, each revealing distinct characteristics of the hydrogel materials. The AFM topographies of the CK-2, W-1, W-2, W-3, W-4, and W-5 hydrogels are depicted in Fig. 5. A notable observation was that the incorporation of watermelon rind led to a significantly rougher surface texture in the hydrogels as compared to those without watermelon rind addition. Upon the addition of watermelon rind to the hydrogel matrix, both height and roughness values were observed to augment, indicative of the successful integration and interaction between the rind components and the polypropylene and polyacrylamide constituents during the hydrogel synthesis process.

### 3.4 Thermogravimetric analysis

**Fig. 6:**
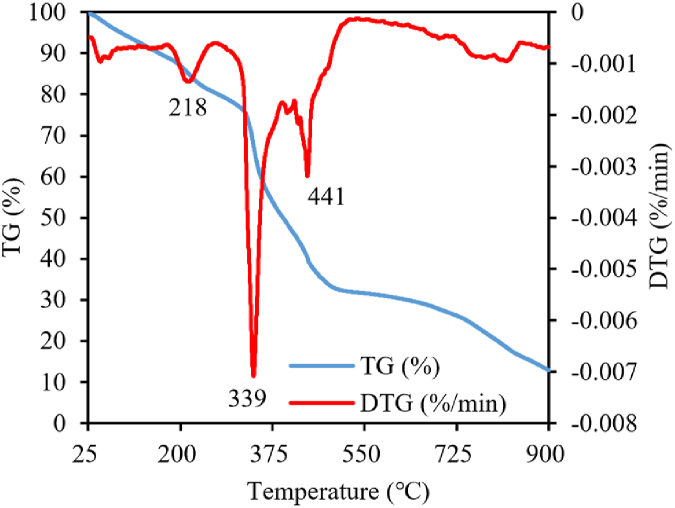
TG and DTG analysis of W-2 treated hydrogels.

The W-2 treated hydrogel with the highest water absorption multiplicity was selected for thermogravimetric analysis as shown in Fig. 6. It is obvious from the DTG curves of W-2 that there are three distinct weight loss phases, 200–240, 324–368, and 424–449, with the weight loss rates of 4.93 %, 20.01 %, and 6.43 %, respectively. The weight loss in the first stage was mainly due to the loss of bound water in the hydrogel [16]. The weight loss in the second stage was mainly due to the decomposition of the organic structure [36]. The weight loss in the third stage was mainly due to the combustion of carbon and other organic matter (including carbonaceous, fructose, etc.) which released some volatile substances [37]. The aforementioned thermogravimetric analysis revealed that W-2 exhibits a decomposition onset temperature exceeding 200 °C, thereby substantiating its robust thermal stability. Consequently, this property ensures the material's suitability for applications under room temperature environment.

### 3.5 Analysis of water absorption and retention properties and reusability of hydrogel

**Fig. 7:**
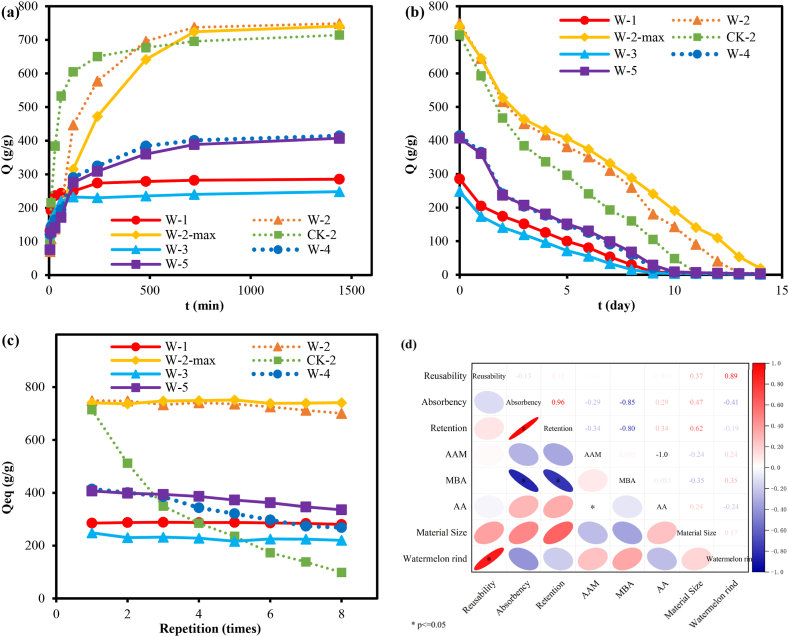
Water absorption and retention curves of hydrogels (a) (b); Qeq of hydrogels after reuse (c); Correlation analysis chart (d). Note: Values are means (n = 3), ∗ denote significant differences among the various treatments at P < 0.05.

The water absorption of the hydrogel at different times is shown in Fig. 7 (a). In this study, the hydrogels were ground into different sizes of particles, <0.25 mm for small powder particles and 0.25–2 mm for large powder particles (W-2-max). From the figure, it could be seen that the equilibrium swelling ratio of W-2 and W-2-max were almost identical. However, the swelling ratio of W-2 reached 447 g/g at 120 min, while that of W-2-max was only 315 g/g at 120 min. It was not until 12 h that the swelling ratios of W-2 and W-2-max reached the same level. The Qeq of W-1, W-2 and W-3 were compared by controlling the constant ratio of AAm and AA additions (3 : 7 for AAm: AA) and varying the amount of MBA added (0.025–0.1 wt % for MBA additions). It can be seen that the lower concentration of MBA (cross-linking agent) could make the Qeq of the hydrogel higher, and the Qeq of W-2 could be as high as 749 ± 32 g/g, which is higher than the swelling ratios of the hydrogel materials prepared by the previous authors at 604 g/g [38], 280 g/g [39], 342 g/g [40],511 g/g [41],253 g/g [42] (Table 3). W-2 had only half of the amount of MBA added to W-1, while the Qeq increased by 162.3 %. W-3 had twice as much MBA as W-1, while the Qeq decreased by 13.0 %. When the amount of crosslinker addition was controlled, and the ratio of AA to AAm was changed (MBA addition of 0.05 wt %, AA: AAm of 7:3, 1:1, and 3:7, respectively), the Qeq of W-4 and W-5 was 42.0 % and 34.2 % higher than that of W-1, respectively. By comparing the Qeq of CK-2 (without watermelon rind added) and W-2 (with watermelon rind added). It was found that the Qeq of CK-2 was slightly lower than that of W-2. Moreover, the water absorption speed of CK-2 is higher than that of W-2, so CK-2 will reach the equilibrium swelling rate faster than W-2.

**Table 3:** Comparison of (AA-co-AAm)/WR hydrogels with similar compounds.

Sr. no.	Reference	Monomer used	Backbone material	Weight ratio (monomer:backbone)	Cross-linker used	Water absorbing capacity (g/g)
1	This research	Acrylic acid; Acrylamide	Watermelon rind	2:1	MBA	749
2	Guo et al., 2022 [38]	Acrylic acid	Cellulose-based	3:1	AMPS	604
3	Meng et al., 2019 [39]	Acrylic acid	Red liquor	5:2	MBA	208
4	Abhisekh et al., 2021 [40]	Acrylic acid	Natural fbre	4:1	MBA	342
5	Zhang et al., 2015 [41]	Acrylic acid	Starch Zeolite 4A	10:1	MBA	511
6	Xiao et al., 2017 [42]	Acrylamide	Starch	1.5:1	MBA	253

Note: MBA is N, N′-methylene bisacrylamide; AMPS is methylpropanesulfonic acid.

The water retention curves of the hydrogels are shown in Fig. 7 (b). With time to the 9th day, the Q of hydrogels except W-2, W-2-max and CK-2 decreased to below 30 g/g. However, the Q of W-2 could still reach 180 g/g.

The Qeq of the hydrogel after repeated utilization is shown in Fig. 7 (c). It was obvious from the figure that CK-2 had a simpler polymer structure due to the formation of the polymer without the addition of watermelon rind, and thus the Qeq of CK-2 was only 13.80 % of that of the 1st use after 7 times of reuse. On the other hand, the hydrogel with watermelon rind had a more complex polymer structure with a 3D network structure, and thus the Qeq did not change significantly in multiple reuses. After eight cycles of intensive use, the WR hydrogel maintained 94.88 % of its initial swelling capacity.

Combined with Fig. 7 (a) and (c). Although the large-size hydrogel absorbed water more slowly, its durability was slightly higher than that of the small-size hydrogel.

#### 3.5.1 Correlation analysis

The correlation analysis between different indicators is shown in Fig. 7 (d). The results indicate a significant positive correlation (P < 0.05) between the amount of watermelon rind added and the reuse rate, as well as between the quantity of MBA reagent added and the water absorbency and water retention of the hydrogel. This further proved that the reusability of the hydrogel prepared in this study was significantly improved with the addition of watermelon rind. What can also be noted from the data in the figure is that MBA has a significant negative correlation (p < 0.05) with water absorption and water retention.

#### 3.5.2 Structural equation modeling and random forests

**Fig. 8:**
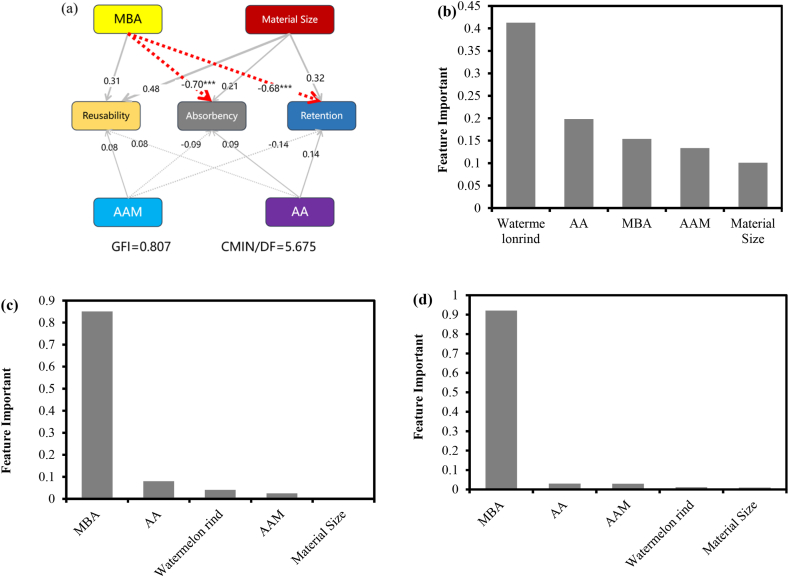
Structural equation modeling (a); Random Forest Model Importance Statistics for Material Reusability (b), Material Absorption (c), and Material Water Retention (d). Note: ∗∗∗ represent 0.1 % significance levels, respectively.

The structural equation modeling of the effect of different reagent material additions and material size on water absorption, water retention, and reusability is shown in Fig. 8 (a). The thickness of the lines represents the standardized coefficients, the solid line represents a positive relationship, the dashed line represents a negative relationship, the red line represents a significance level of 0.1 %. From Fig. 8 (a), it could be seen that MBA additions had a significant negative relationship with water absorption and water retention (P < 0.001), which was also reflected in Fig. 8 (c) (d) that MBA had the greatest effect on water absorption and water retention.

The significance calculation of the reusability of the hydrogels using the random forest model is shown in Fig. 8 (b), where the addition of watermelon rind has the greatest effect on the reusability.

Based on the above conclusions, W-2, which is optimized for water absorption and water retention, was used in the subsequent experimental materials.

### 3.6 Analysis of Qeq of hydrogels at different pH equilibria

**Fig. 9:**
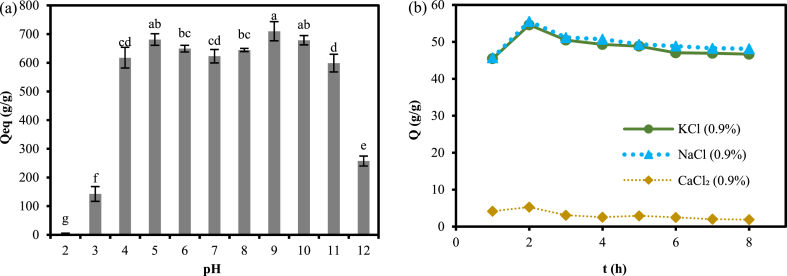
Equilibrium swelling ratio of hydrogels at different pH (a), Swelling ratio of hydrogel in different salt ion solutions (b).

The Qeq of the hydrogel at different pH solutions is shown in Fig. 9 (a). It could be seen that Qeq increased rapidly when pH was increased from 2 to 4, followed by more stability in the range of 4–10, and then Qeq decreased sharply when pH was increased to 12. First, both polyacrylic acid and polyacrylamide contain functional groups that can be ionized. Polyacrylic acid contains a carboxyl group (-COOH), while polyacrylamide contains an amide group (-CONH_2_). In an acidic environment, the carboxyl group is easily ionized to carboxyl ions (-COO-), while the amide group is not easily ionized. When pH = 2–4, the higher concentration of H^+^ in solution makes the carboxyl groups of polyacrylic acid easily ionized to form carboxyl ions and enhances the hydrogen bonding interactions in the polymer network structure, producing additional physical cross-linking, which increases the water uptake capacity of the hydrogel, resulting in a rapid increase in Qeq [43]. When the pH was in the range of 4–10, the H^+^ concentration gradually decreased, and the ionization of the carboxyl ions gradually decreased, but the (-COO-) and carboxyl groups formed a buffer system in the three-dimensional network structure formed by the polymer [44]. Thus Qeq is relatively stable. However, when the pH was increased to 12, the solution became alkaline, the H^+^ concentration was very low, and the ionization of carboxyl ions was greatly reduced; at the same time, the OH- concentration increased, and the rapid increase of Na^+^ on (-COO-) weakened the repulsive force between anions, which affected the structure and water-absorbing capacity of the hydrogel, causing Qeq to decrease sharply. It could also be found that the Qeq of the hydrogel was not maximized at a solution pH of 7 but peaked in acidic or alkaline environments (pH = 5, 9, 10).

### 3.7 Analysis of the swelling ratio of hydrogels in solutions of different salt ions

As can be seen from Fig. 9 (b) The swelling ratio of the hydrogel in a 0.9 % Ca^2+^ solution was almost 0. Also, the swelling ratio in K^+^ and Na^+^ solutions was much smaller than that in pure water, ranging from about 45 g/g to 55 g/g. There was no significant difference (P < 0.05) between the swelling ratios of the hydrogels in K^+^ and Na^+^ solutions.

### 3.8 Research on water retention and water holding capacity of soil

**Fig. 10:**
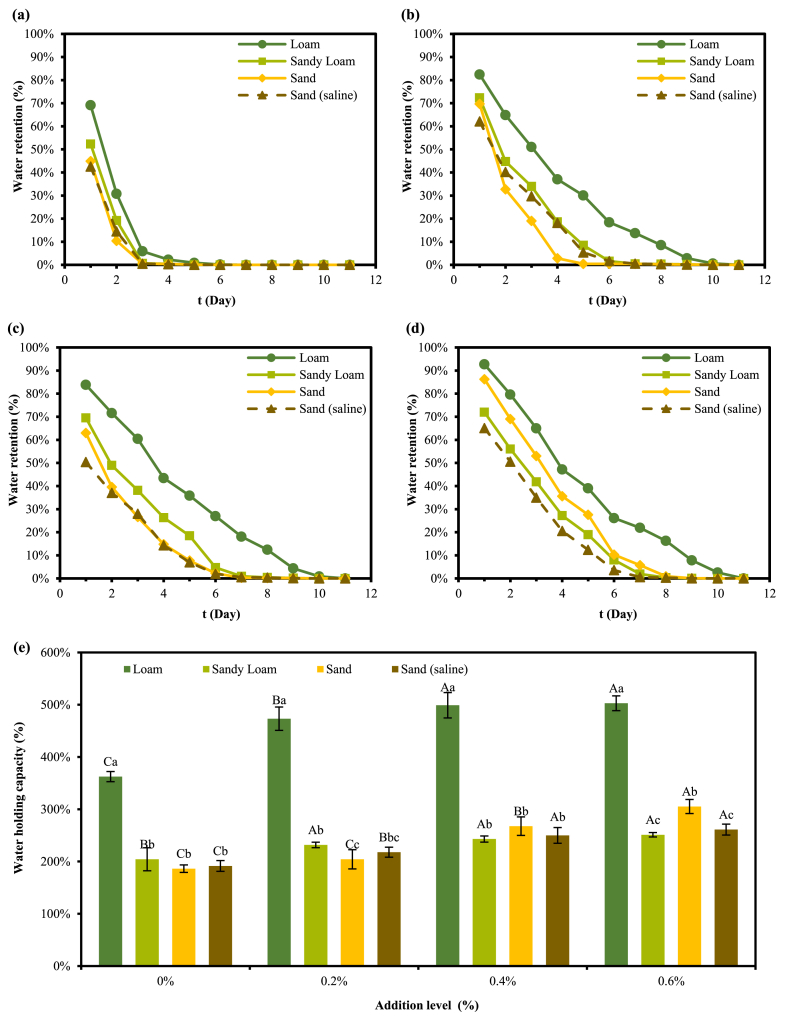
Water retention curves of different soils at 0 % (a), 0.2 % (b), 0.4 % (c), 0.6 % (d) hydrogel additions; water holding capacity of different soils at different hydrogel additions (e). Note: Capital letters indicate significance of different hydrogel addition gradients and lowercase letters indicate significance between different soil types (P < 0.05).

The effects of different additions on water retention of different soils are shown in Fig. 10(a)–(d). It could be seen that the water retention properties of soils other than sand were significantly improved by the addition of hydrogel to different soils. It was worth noting that the increase in water retention of the hydrogel from 0 % to 0.2 % was more significant than that of 0.2 %–0.4 % and 0.4 %–0.6 %. This indicated that the 0.2 % addition was more cost-effective. Combined with Fig. 10 (e), it could also be found that the loam had the greatest increase in water holding and water retention capacity with the addition of hydrogel.

From Fig. 10 (e), It could be seen that loam held water better at hydrogel additions of 0.4 % and 0.6 %, sandy loam held water better at hydrogel additions of 0.2 %, 0.4 %, and 0.6 %, sand held water better at a hydrogel addition of 0.6 %, and sand (saline) held water better at hydrogel additions of 0.4 % and 0.6 %. The increase in water holding and water retention with increasing hydrogel addition was more pronounced in sand than in sandy loam and sand (saline). Water retention and water-holding capacity of sandy loam were the lowest at 0.2 % hydrogel addition. However, when the gel addition was increased to 0.6 %, the water retention and water-holding capacity of the sandy loam exceeded that of the sandy loam (saline) and sandy loam.

### 3.9 Germination of barley under drought stress

**Fig. 11:**
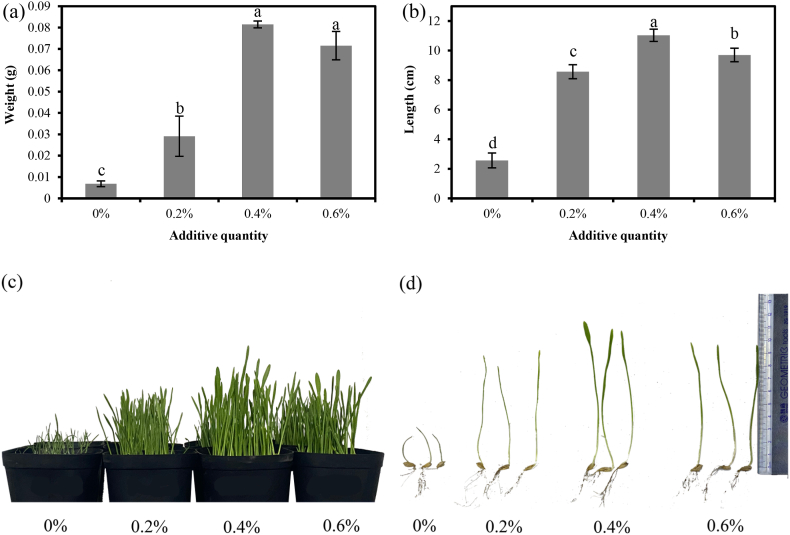
Weight (a), height (b), overall growth (c), and schematic diagram (d) of barley seedlings with different hydrogel additions. Note: Different lowercase letters indicate significance at p < 0.05.

The weight and height of barley seedlings in the above-ground portion are shown in Fig. 11 (a)(b), and the best growth of barley was observed when the addition amount was 0.4 % (35 % FWC). The average weight of barley seedlings at 0.4 % hydrogel addition was 1092 %, 180 %, and 13.9 % higher than those at 0 %, 0.2 %, and 0.6 % addition, respectively. The average height of barley seedlings at 0.4 % hydrogel addition was 330 %, 28.8 %, and 13.7 % higher than at 0 %, 0.2 %, and 0.6 % addition, respectively. Combined with Fig. 11 (c)(d), it could be seen that the addition of hydrogel significantly improved the growth of barley seedlings under drought stress. This was similar to the results of previous studies [45]. However, adding too much hydrogel could be counterproductive, with barley growing worse at 0.6 % hydrogel than at 0.4 %.

### 3.10 Adsorption studies of MB dye

**Fig. 12:**
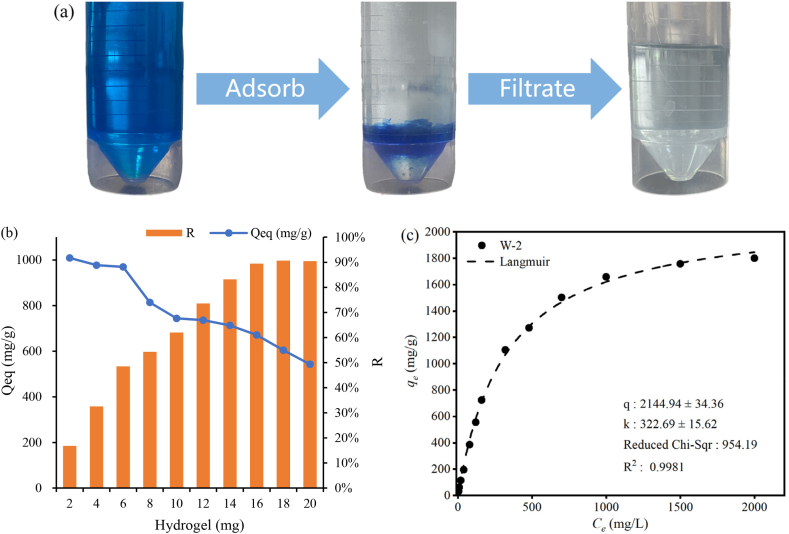
Adsorption of MB by WR hydrogel (a), effect of WR hydrogel addition on MB removal (b), adsorption values fitted with the Langmuir model (c).

The adsorption of MB by WR hydrogel is shown in Fig. 12(a). It can be seen that the addition of WR hydrogel to the MB solution can absorb MB inside the solution, making the color of WR hydrogel obviously darker. After filtration the color of the solution becomes significantly lighter. As shown in Fig. 12(b), When the MB solution was 25 ml at a concentration of 480 mg/L, only 18 mg of WR hydrogel was added, and the dye removal rate could reach more than ninety percent (90.67 %). In Fig. 12(c), the adsorption value of MB dye by WR hydrogel can be well fitted by using Langmuir model, and the R^2^ is 0.9981. 5 mg of WR hydrogel was added to 2000 mg/L MB solution (pH = 8) and equilibrated for 12 h, and its qe was 1800.08 mg/g. Using the Langmuir model, the theoretical maximum adsorption amount of WR hydrogel was 2144.94 mg/g.

## 4 Discussion

Water scarcity poses a significant global challenge exacerbated by factors such as climate change, population growth, and inefficient water utilization, notably in agriculture. Superabsorbent hydrogel materials present a promising solution by efficiently absorbing, retaining, and releasing substantial water volumes. This capability enhances water use efficiency, improves soil structure, promotes plant growth, and reduces the need for frequent irrigation. However, the knowledge about the use of watermelon *(Citrullus lanatus)* rind (WR) to develop superabsorbent hydrogels and its potential to improve barley germination under drought conditions needs to be highlighted. This discussion aims to explore the broader implications of the research findings, underscore the importance of employing watermelon rind as a base material, assess the influence on soil characteristics. The WR hydrogel synthesized in this study represents a significant innovation in utilizing organic waste and enhancing water-holding capacities in agricultural soils, offering a sustainable solution to water scarcity challenges.

### 4.1 Characterisation of hydrogels

The characterisation of the hydrogel shows that the N-H comes from the addition of AAm and MBA reagents to the hydrogel. Further evidence that the hydrophilic amide group in the reagent binds to the material. All of these polyamide molecules contain (O = )-C-NH-, and the main characteristic bands in their spectra are all related to this group. The carboxyl group also plays a key role as a potent hydrophilic group in the water absorption process [[46], [47], [48]]. The absorption peak at 1170 cm-1 attributed to the asymmetric oxygen bridge (C-O-C). The absorption peak at 1030 cm-1 attributed to the C-O stretching vibration in the alcohol hydroxyl group of watermelon rind was almost invisible after the reaction. This indicates that MBA acts as a cross-linking agent during hydrogel synthesis, and APS acts as an initiator, which leads to the breakage of the glycosidic bond of watermelon rind and the generation of macromolecular radicals under the initiating action, allowing acrylic acid and acrylamide to be grafted on the end groups after the breakage of the glycosidic bond. From the characteristic absorption peaks of amide and carboxyl groups of FTIR spectra, it can be concluded that (AA-co-AAm)/WR hydrogel were successfully synthesized with the presence of MBA as a cross-linking agent, in which the chain structure of the polymers has a strong water-absorbent property, which attracts the water to enter into the material and forms hydrogen bonds through the action of hydroxyl, carboxyl, and amide groups among the polymers with water molecules, to uniformly distribute the water within the material. The SEM analysis shows that the rough surface of the hydrogel with the addition of watermelon rind indicated the formation of a well-connected complex three-dimensional network structure with an inhomogeneous surface cross-linking pattern. The larger surface of the hydrogel allowed water molecules to enter more easily and attach to the hydrophilic groups, thus increasing the water absorption of the hydrogel [36]. The addition of more cross-linking agents can make the material more tightly connected, but at the same time, it may reduce its swelling ratio [49].

Analysis of the water absorption and retention properties and reproducibility of the hydrogels reveals that the size of the hydrogel had less effect on the equilibrium swelling ratio but more effect on the speed of water absorption. This result was in agreement with the results of previous studies [15]. The more MBA is added, the lower the water absorption rate is [15]. The reason for this phenomenon may be that a high crosslink density limits the limits of hydrogel expansion. In this study an appropriate reduction of AA: AAm can improve the water absorption rate. However, some studies showed that the water absorption rate was rather reduced after lowering AA: AAm [15]. This may be due to the differences in the structure of the hydrogels caused by the different choices of organic wastes, which in turn resulted in different water absorption multiplicities of the hydrogels. The reason why hydrogels without the addition of watermelon rind reach the equilibrium swelling rate faster is because cellulose and pectin in watermelon rind played an important role in the preparation of hydrogels. They generated compounds containing carboxyl and hydroxyl groups during the reaction process, and these compounds could react with acrylic acid (AA) and acrylamide (AAm) to form a more complex polymer structure. This complex polymer structure increased the crosslink density of the hydrogel, thereby reducing its water absorption rate. When watermelon rind was not added, acrylic acid (AA) and acrylamide (AAm) directly underwent a free radical polymerization reaction, forming a relatively simple polymer structure with a lower crosslink density. Therefore CK-2 will reach the equilibrium swelling ratio faster compared to W-2.

In this study the W-2 treatment added the least amount of MBA reagents and had a lower crosslink density, but even with the lower crosslink density, the W-2 hydrogel maintained a high water retention capacity. However, it has been shown that while low crosslinker addition increases the maximum water absorption multiplicity of the hydrogel, it may at the same time reduce the structural strength of the hydrogel [50]. Therefore, only by appropriately reducing the MBA can the water retention capacity be improved while increasing the water absorption rate. In future research, we need to find the optimal reagent addition ratio for the water absorption, water retention, and reusability of hydrogels. It could also be seen that the curve of CK-2 was steeper, indicating that the synthetic hydrogel lost water faster in the absence of pectin and cellulose in the watermelon rind. Since the structure of the hydrogel polymeric aggregate with the addition of watermelon rind was a more complex three-dimensional network structure, after eight cycles, the (AA-co-AAm)/WR hydrogel retained 94.88 % of its initial swelling capacity, surpassing the (AA-co-AAm) hydrogel without WR, which retained only 13.80 %. In this way, it was proven that the addition of watermelon rind in our developed hydrogel was important for material durability. This, in turn, showed the practical significance of our hydrogel prepared from watermelon rind as organic waste.

### 4.2 Effect of different pH and salt ion concentrations on the Qeq of hydrogels

WR hydrogels have a weaker Qeq in strong acid and alkali environments. The reasons for this phenomenon are related to the number of hydrogen bonds in the polymer network and the degree of ionization of the hydrogel [51]. It could also be found that the Qeq of the hydrogel was not maximized at a solution pH of 7 but peaked in weakly acidic or alkaline environments (pH = 5, 9, 10). This was similar to the results of previous studies [37]. The reason for this phenomenon may be that when the pH increases from 5 to 7, the concentration of H^+^ in the solution decreases and the degree of ionization of the carboxyl group may decrease, leading to a decrease in the Qeq value. When the pH increases from 7 to 9, some other chemical reactions may take place, such as the amide group may start to ionize, or the ionization of the carboxyl group may start to increase, which may lead to a renewed increase in the Qeq value. However, the real reason needs to be further investigated.

By studying the swelling of hydrogels in salt solutions, It was found that salt ions in high valence states had the greatest effect on hydrogel swelling, which might have been related to the chelating ability of the ions. The monovalent and divalent cations differed in their ability to form stable chelates with anions. Divalent cations were more likely to form stable chelates with -amino groups on highly absorbent polymers due to their higher charge density, allowing better formation of stable chemical bonds with anions. Additionally, the ion's radius was an important factor in the water absorption capacity of superabsorbent hydrogels. The larger the radius of the ion, the lower its charge density, and the smaller the shielding effect on the anionic group. This increased the chances of collision and binding between cations and anions, making it easier for anions to react with cations and less likely to enter the polymer network. Thus, it reduced the water absorption of superabsorbent polymers [36,52]. However, there was no significant difference in the swelling rate of hydrogels in K^+^ and Na^+^ solutions in this study. It is inconsistent with the conclusion that the ionic radius affects the water absorption of hydrogels as mentioned by the previous authors. It is possible that the effect of different valence states on the water absorption of hydrogels is much larger than that of ionic radius, so that the difference in the water absorption of hydrogels between K^+^ and Na^+^ ions in this study is not obvious.

### 4.3 Effect of hydrogel on water retention properties of soil and adsorption of dye in water

The incorporation of hydrogels can significantly enhance the water retention capabilities of soils, with particularly notable improvements observed in loam. This was because the loam used in this experiment was purchased from the market potting soil, the organic carbon content of soil was about 27.1 %, and a large amount of organic material made the soil bulk density very low. The low bulk-density soil had larger voids, which were more favorable for the expansion of the hydrogel. The increase in water holding and water retention with increasing hydrogel addition was more pronounced in sand than in sandy loam and sand (saline). This was due to the high bulk density and fractal dimension of the sandy loam selected for the study. The high fractal dimension number implied irregularity in soil particle size, where voids between large particles were filled by small particles, making the soil porosity relatively smaller. In contrast, the fractal dimension and bulk density of sand were lower than that of sandy loam, and the hydrogel was more likely to swell in the pores of soil particles. Therefore, the water-holding and water-retaining properties of sand improved more with hydrogel addition than with sandy loam. While sand (saline-alkaline) had a large number of salt ions, according to the conclusion obtained above, salt ions would reduce the swelling ratio of hydrogel, so the enhancement of water-holding and water-retention properties of hydrogel in the sand (saline-alkaline) was smaller compared to sand [15]. In conclusion, the addition of hydrogel in this study can alleviate the problem of poor water-holding and water-retaining capacity of soils in Northwest China to a certain extent, and then improve the crop yield.

Moisture is vital for the growth of young crop seedlings. Seed growth is more affected when soil moisture falls below 10 percent [53]. In this study, the efficacy was evaluated on barley (Hordeum vulgare) through a pot experiment using different hydrogel concentrations (0 %, 0.2 %, 0.4 %, and 0.6 %) under 35 % FWC water-holding capacity drought stress. Results demonstrated that the optimal hydrogel addition ratio of 0.4 % significantly enhanced shoot biomass and shoot length of barley by compared to the control, showcasing its effectiveness in improving barley germination during drought conditions. However, the addition of too much hydrogel can be counterproductive. This may be due to the fact that too much hydrogel absorbs too much water, making the soil too wet, which may lead to insufficient oxygen supply to the plant roots, affecting root respiration, and may even lead to root rot. Also too much hydrogel may change the physical structure of the soil, making it too compact and affecting the growth and expansion of plant roots.

### 4.4 Adsorption potential of methylene blue by watermelon rind hydrogel

WR hydrogel can also significantly adsorb MB dye in water. WR hydrogel has a three-dimensional network structure, and its surface contains hydrophilic functional groups such as carboxylic acid (-COOH) and amino (-NH^2^), which are capable of forming chemisorptive interactions with MB, including ion exchange, electrostatic attraction, and complexation [54]. Moreover, this loose macroporous mesh structure of WR hydrogel after absorbing water increases the effective contact area between the hydrogel and the dye, which is favorable for the adsorption of the dye by the hydrogel [55]. The theoretical maximum adsorption amount of MB on WR hydrogel was 2144.94 mg/g using Langmuir model, which was much larger than that of 1800.08 mg/g in the experiment, indicating that there is still a large adsorption potential of MB on WR hydrogel. In the future, the adsorption of MB dyes on WR hydrogel can be further investigated to improve the removal efficiency of MB dyes on hydrogel, so as to make a greater contribution to environmental protection and sustainable development.

### 4.5 Challenges and future directions

The utilization of watermelon rind as a raw material for hydrogel synthesis is a notable aspect of this study. Watermelon is a widely cultivated crop, and the rind, often discarded as waste, represents a significant source of biomass. By repurposing this waste product, the study not only contributes to waste reduction but also paves the way for the development of eco-friendly and biodegradable materials. The high cellulose and pectin content in watermelon rind likely contributes to the hydrogels' enhanced water absorption and retention properties, making it a valuable resource for hydrogel production.

## 5 Conclusion

In this study, superabsorbent hydrogels prepared from watermelon rind as agricultural waste. The equilibrium swelling capacity at optimum reagent addition could reach 749 ± 32 g/g.

The incorporation of the WR hydrogel into various soil types enhanced the water-holding and water-retention capacities, with potting soils rich in organic matter yielding the best outcomes.

The hydrogel significantly bolstered the growth of barley seedlings under conditions of drought stress (35 % FWC) by improving the soil's water retention capacity, with an optimal hydrogel addition rate of 0.4 %.

WR hydrogel has significant adsorption effect on MB dye, only need to add 18 mg WR hydrogel on 25 ml 480 mg/L MB solution can reach 90.67 % removal rate.

A 5 mg WR hydrogel equilibrated in 2000 mg/L MB solution (pH = 8) for 12 h resulted in a *q*_*e*_ of 1800.08 mg/g. The maximum adsorption was found to be 2144.94 mg/g by Langmuir model.

## Data availability

No data was used for the research described in the article.

## Funding

The authors thankfully acknowledge the financial support from the 10.13039/501100007932Gansu Agricultural University Public Recruitment Doctoral Research Start-up Fund (No. GAU-KYQD-2018-39), 10.13039/501100004775Natural Science Foundation of Gansu Province (No. 20JR10RA543, 21JR7RA811), and Gansu Outstanding Graduate Student Innovation Star Program (No. 2023CXZX-694).

## CRediT authorship contribution statement

**Bingqin Teng:** Writing – original draft, Methodology, Investigation, Data curation. **Yuan Zhong:** Formal analysis, Data curation, Conceptualization. **Jun Wu:** Project administration, Funding acquisition. **Jiachen Zhu:** Visualization, Methodology. **Liqun Cai:** Resources, Conceptualization. **Peng Qi:** Software, Investigation. **Zhuzhu Luo:** Software, Investigation.

## Declaration of competing interest

The authors declare that they have no known competing financial interests or personal relationships that could have appeared to influence the work reported in this paper.
